# Intersectionality-Informed HIV Cure-Related Research at the End of Life: A Call to Action

**DOI:** 10.3390/ijerph23030295

**Published:** 2026-02-27

**Authors:** Ali Ahmed, Brittany Shelton, Malachi P. Keo, Kris H. Oliveira, Alejandra Mortlett-Paredes, Whitney Tran, Samuel O. Ndukwe, Jeff Taylor, Thomas J. Villa, Bridgette Picou, Leslie D. Matherne, Renato Bobadilla-Leon, Rachel Lau, Stephanie Solso, Cheryl Dullano, Davey Smith, Antoine Chaillon, Robert Deiss, Sara Gianella, Karine Dubé

**Affiliations:** 1 Division of Infectious Diseases and Global Public Health (IDGPH), School of Medicine, University of California San Diego (UCSD), La Jolla, CA 92093, USArbobadillaleon@health.ucsd.edu (R.B.-L.);; 2 Department of Public Health, University of Tennessee Knoxville, Knoxville, TN 37996, USA; 3 Department of Neurology, Massachusetts General Hospital of Harvard Medical School, Boston, MA 02114, USA; 4 Departamento de Infectologia e Medicina Tropical, Faculdade de Medicina, Universidade de São Paulo, São Paulo 05403-000, Brazil; 5 Department of Neurosciences, School of Medicine, University of California San Diego (UCSD), La Jolla, CA 92093, USA; amorlettparedes@health.ucsd.edu; 6 HIV+ Aging Research Project—Palm Springs, Palm Springs, CA 92264, USA; 7 HOPE Martin Delaney Collaboratory for HIV Cure Research Community Engagement, Washinton, DC 20004, USA; 8 The Well Project, Palm Springs, CA 92262, USA; 9 Quillen College of Medicine, East Tennessee State University, Johnson City, TN 37614, USA; 10 AntiViral Research Center (AVRC), University of California San Diego (UCSD), San Diego, CA 92103, USA

**Keywords:** HIV cure research, end of life, intersectionality, justice, equity, diversity, inclusion, accessibility, people with HIV

## Abstract

**Highlights:**

**Public health relevance—How does this work relate to a public health issue?**

- End-of-life HIV cure-related research can advance discovery while honoring dignity and legacy, but participation remains demographically skewed, reinforcing inequities in who is informed, invited, and supported.
- Drawing on eight years of UC San Diego Last Gift experience, literature review and community engagement, this paper offers a practical approach to embed intersectionality-informed justice, equity, diversity, inclusion, and accessibility throughout end-of-life study design and delivery.

**Public health significance—Why is this work of significance to public health?**

- The paper applies an intersectionality-informed framework to convert ethical guidance into practical procedures organized across three themes and eight domains, designed to be implemented directly or adapted to local capacity.
- We frame equity as integral to methodological rigor that strengthens trust, representation, and the overall validity of HIV cure research findings.

**Public health implications—What are the key implications or messages for practitioners, policy makers and/or researchers in public health?**

- Practitioners and researchers can help protect participant autonomy by removing study design barriers that limit or pressure participation, using multi-session consent with teach back, establishing transparent proxy pathways, and keeping research separate from clinical care.
- Policymakers and funders can advance accountability by requiring transparent reporting and real-time course correction tools, including enrollment dashboards that track representation and guide corrective action.

**Abstract:**

**Introduction:** End-of-life (EOL) HIV cure-related research offers a unique opportunity to advance scientific discovery while honoring the values, dignity, and legacy of people with HIV. However, participation remains demographically skewed, mirroring long-standing inequities in who is informed, invited, and supported to take part. Synthesizing eight years of experience, published literature reviews, and community engagement from the University of California San Diego’s Last Gift program, we propose strategies to embed justice, equity, diversity, inclusion, and accessibility (JEDIA) throughout the design and implementation of EOL HIV cure-related studies. **Discussion:** Using intersectionality as a structural analytic framework, we examine how interlocking systems and social determinants shape access, consent, and participant experience, and we translate ethics into action across three themes and eight domains. As examples, we facilitate equitable access by implementing solutions that address gaps limiting awareness and feasibility of participation. We establish ongoing consent through multi-session consent processes with teach-back methods, clear healthcare proxy pathways, and explicit separation of research activities from clinical care. We center lived experiences by partnering with people with HIV and community groups, customizing participation, and honoring cultural and spiritual needs. We enable real-time course correction by using a dashboard that monitors enrollment patterns and representation. **Conclusions:** An intersectionality-informed, participant-centered approach is both feasible and essential to ensure HIV cure-related research advances with fairness, trust, and global relevance. Programs such as the Last Gift show that scientific rigor, integrity, and participant dignity can coexist, establishing a model for equitable HIV cure discovery.

## 1. Introduction

Lifelong antiretroviral therapy (ART) remains necessary for people with HIV (PWH), and durable ART-free control or cure is still out of reach [1,2]. The International AIDS Society (IAS)’s 2021 Global HIV Strategy calls for accelerating discovery while embedding social and ethical responsibility across the research lifecycle [3]. End-of-life (EOL) HIV cure-related studies uniquely enable access to otherwise unattainable tissues and reservoirs and are feasible when implemented with robust safeguards and recognition of participant contributions [4]. EOL HIV cure-related research encompasses a range of activities that vary in intensity and ethical and operational requirements [4,5]. These may include ante-mortem observational assessments and biospecimen collection, minimally invasive tissue sampling, post-mortem tissue donation such as rapid autopsy and, in some contexts, interventional studies conducted near the end of life [4,6,7,8]. Because these categories differ in participant burden, procedural complexity, and support needs, careful operational considerations and ethical oversight are essential. Drawing on original and existing empirical ethics research, the University of California San Diego (UCSD)’s Last Gift program provides guidance for respectful recruitment, ongoing informed consent, and healthcare proxy/loved one engagement [5,9]. With such protection, participants and their families often describe altruistic and legacy-related motivations, alongside acceptability of participation [10].

Current participation in HIV cure-related research reflects persistent inequities in who is invited, supported, and empowered to take part. Treatment Action Group (TAG)’s latest audit, covering results presented or published from 2018 through mid-2025, identified 161 HIV cure-related studies enrolling 7792 participants [11]. Among the 7682 individuals with data reporting sex or gender, only 19.2% were women, 0.4% were transgender women, 0.04% were transgender men, and 0.1% were non-binary [11]. This contrasts with global epidemiology where women make up 51% of the population with HIV [12], and transgender women, who represent less than 2% of the population, are 48 times more likely to be diagnosed with HIV, with a pooled HIV prevalence of 19% [13].

Racial and ethnic representation is similarly lacking [14]. Among 5300 individuals with data reporting race or ethnicity, only 18% were Black, 7.3% identified as Hispanic or Latino, and 6.6% were Asian [11,15]. Forty-seven studies provided no race or ethnicity data, and five did not report sex, gender, or race at all [11]. Globally, individuals of African descent bear the highest HIV burden, with approximately 64% of the world population with HIV residing in Africa [12].

People who inject drugs (PWID) are another key study population. The Joint United Nations Programme on HIV/AIDS (UNAIDS) reports that in 2022, the relative risk of acquiring HIV was 14 times higher for PWID than for people in the overall adult population, and that the global median HIV prevalence among PWID is 5.0%, ranging from 0% to 32% across 47 reporting countries, compared with an HIV prevalence of 0.7% among the total global adult population aged 15 to 49 years [16]. Despite this, PWID are significantly underrepresented in HIV cure-related research.

Most cure-related studies are from North America or Europe, with limited reporting of socioeconomic status, sexual orientation, migratory status, and other social determinants. This lack of demographic data restricts the relevance of findings to the broader global HIV community [11,15]. As a result, HIV cure-related research reflects a narrow segment of PWH, leaving many communities underrepresented despite bearing a significant share of the global burden [17].

Demographic representation is an ethical, scientific, and epistemological necessity [2]. Ethically, it determines which communities shape, interpret, and inform HIV science [18,19]. Scientifically, broader participation enhances understanding of reservoir heterogeneity, reduces the risk of effect modification and improves external validity [20]. Therefore, it is essential research that reflects the complete clinical and social diversity of people with HIV, including PWID, transgender and gender-diverse (TGD) individuals, and racial/ethnic minorities [17,21].

To guide critical reflection, we use intersectionality [22,23] as an analytical framework that examines how multiple social positions such as gender, race, class, sexuality, disability, and geography interact with systems of power, including patriarchy, coloniality, racism, and neoliberal political economy [24,25]. Rather than treating these dimensions as descriptive covariates, an intersectional approach reveals how social hierarchies shape participation, ethical norms, and the production of biomedical knowledge, while also prioritizing the transformation of these power structures [22,26]. In the context of EOL HIV cure-related research, embedding intersectionality means recognizing that inequalities are structurally produced through institutions, research agendas, and funding mechanisms [27], and adopting participatory and reflexive approaches that center the lived experiences and contributions of people most affected by HIV [22]. In doing so, justice, solidarity, and inclusion become integral to scientific rigor and ethical practice [22,24,25,27].

Positioned as a practice-informed call to action, we propose a roadmap to embed intersectionality, justice, equity, diversity, inclusion, and accessibility (JEDIA) in HIV cure-related research at the EOL. Intersectionality is not synonymous with JEDIA; JEDIA names equity commitments and implementation priorities, whereas intersectionality is used here as a structural analytic framework to examine interlocking systems of power and the institutional processes that shape participation and outcomes. Our aim is to ensure that scientific progress advances in tandem with dignity, agency, and community benefit, translating empirical ethics lessons into procedures that expand access and strengthen accountability.

## 2. Discussion

This paper distills interdisciplinary lessons from the UC San Diego’s Last Gift HIV cure research program, developed and implemented with continuous community engagement since 2017 [28]. We identified lessons through an iterative synthesis of eight years of program implementation, structured team debriefs and reflexive review of adaptations, ongoing input from Community Advisory Boards (CABs) and ethics committees, and review of empirical ethics, palliative research methods, and community-engaged research guidance. We documented recurring challenges, effective responses, and decision points across these sources and then grouped them into the three themes and eight domains presented below. We did not conduct a formal systematic review; instead, we used this informal but structured approach to transparently ground the recommendations in practice-based evidence and external guidance. We use this synthesis to identify actionable practices that can generalize beyond a single site and inform scalable implementation [8,18,29,30,31,32,33,34,35,36,37]. Implementation also involves real-world tradeoffs. Teams often balance staff capacity with multi-session and ongoing consent practices, budget constraints with navigation and wrap-around supports, and protocol standardization with culturally responsive tailoring. We therefore present these recommendations as a flexible menu that programs can phase in based on local resources, infrastructure, and partnership capacity. We use the Last Gift program as an illustrative case, and we present the domains and underlying principles as broadly transferable while treating specific operational details as examples that may vary by setting.

Over eight years, the Last Gift program has shown that HIV cure-related research at the EOL can ethically engage communities and answer scientifically rigorous questions. However, the translation of findings depends on representative sampling. Despite operating in one of the most demographically diverse regions in the United States (U.S.), enrollment remains disproportionately composed of older White cisgender men. This pattern reflects a broader trend in global HIV cure-related studies, where women make up only 20% of participants, and racial and ethnic minorities are consistently underrepresented (roughly 10%) [6,12]. These enrollment disparities reveal flaws in traditional design checkpoints for achieving ethical contribution and external validity. A priori power calculations and regulatory review are not enough; as a field, without structured feedback and iterative processes, even well-intentioned studies risk perpetuating inequity.

Our analysis centers on the lived experiences of participants and next-of-kin/loved ones, structured team reflexivity, and shared governance with CABs and ethics committees [4]. Although many participants describe altruistic motivations [21,27], altruism does not offset barriers rooted in poverty, housing instability, linguistic exclusion, immigration precarity, and mistrust in research. Equity must be resourced, operationalized, and monitored in real time with explicit lines in budgets, staffing, and oversight structures [38].

We write as a multidisciplinary team of clinicians, socio-behavioral scientists, ethicists, and community partners whose identities span different genders, racial and ethnic backgrounds, sexual orientations, and lived experiences with HIV and caregiving. This heterogeneity models the intersectionality we call for in the field; research conducted for diverse communities must also be conducted with them. Diverse teams strengthen methodological rigor, enhance external validity, and foster trustworthiness—the foundations of ethical HIV cure science [19,20].

Through structured, iterative co-design with community advisors and synthesis of empirical ethics and literature, we identified eight domains within three themes to integrate intersectionality in EOL HIV cure-related research. For each domain, we present our underlying rationale and methodological considerations. We illustrate each domain with examples from the Last Gift program. For example, we set enrollment goals to reflect local HIV epidemiology, use teach-back at predefined points to verify comprehension, document interpreter use or language concordance during consent, and invite families to co-design memorial and stewardship options. These practices comprise a menu to adapt to local capacity and resources. Programs can adopt the core principles without replicating the specific Last Gift workflows. Each aligns with the Good Participatory Practice (GPP) stakeholder engagement framework [39] and with the UNAIDS 2024 terminology [38] guidelines for inclusive, stigma-aware communication. We conclude with an adaptable checklist for research teams, emphasizing that these domains are essential components of rigorous scientific study design. Throughout, intersectionality is applied to identify and address structural barriers and power dynamics, rather than serving as a general descriptor of diversity.

**Figure 1:**
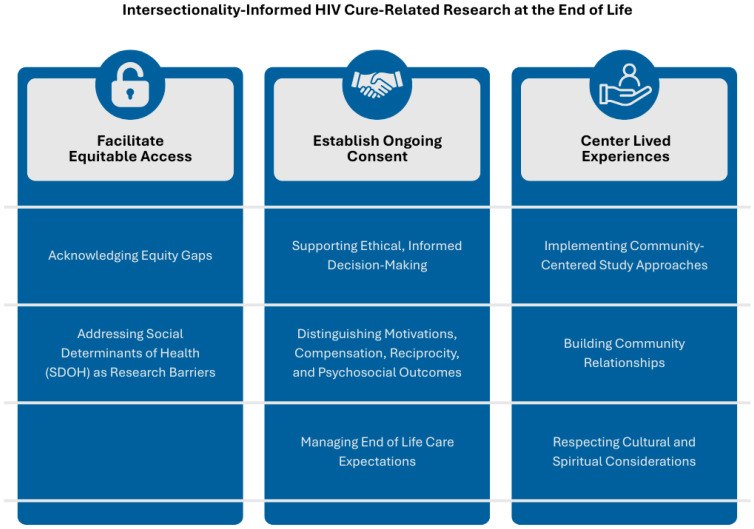
Framework for Intersectionality-Informed HIV Cure-Related Research at the End of Life.

Figure 1 depicts the three interrelated themes and eight domains that structure intersectionality-informed EOL HIV cure-related research. Readers can use the framework as a practical roadmap to connect common study decisions to concrete domains, then select corresponding implementation strategies and example metrics in Table 1. The left to right structure highlights a progression from access, to consent, to lived experiences, while recognizing that domains often operate in parallel and may require iteration over time. The framework also supports prioritization by helping teams identify foundational, broadly transferable practices versus components that may require additional infrastructure, funding, or long-standing community partnerships.

### 2.1. Facilitate Equitable Access

#### 2.1.1. Acknowledging Equity Gaps

EOL HIV cure-related research is operationally feasible and supported by the community when implemented with rigorous safeguards [4,9,10]. From 2017 to 2025, UC San Diego’s Last Gift program enrolled a cohort of approximately 65 volunteers in EOL HIV cure-related research. Most participants identify as cisgender men and as White, with comparatively few women, few transgender and gender-diverse participants, and limited participation from people who identify as Hispanic or Latino [28]. Women’s participation, 11 out of 62 (17.7%), is consistent with local proportions, yet representation of Black or African American and other racially and ethnically diverse groups remains limited [28,40]. Because the Last Gift is an end-of-life tissue donation study, the cohort is expected to skew older, since tissue donation is most feasible among participants who are nearer the end-of-life and who can complete the required procedures within a clinically appropriate window. This structural feature, combined with the realities of time limited grant mechanisms and annual reporting requirements, constrains the extent to which a tissue donation cohort can mirror the age distribution of new diagnoses. Accordingly, the cohort has a higher median age of about 63 years, which differs from local epidemiology, where new diagnoses are most common at ages 30 to 39, even though 58% of PWH in San Diego County are now 50 years or older [40]. As the population of Black, Indigenous, and other people of color (BIPOC) aging with HIV increases locally, the share of BIPOC participants at EOL is expected to grow, and enrollment strategies should anticipate and support that shift [40].

We recommend that research teams apply an intersectionality-informed approach, which centers community co-creation of corrective actions [41]. This framework shifts attention from individual-level explanations for non-participation to system-level mechanisms that differentially distribute information, opportunity, and protection. Persistent structural racism [42] and medical mistrust [43,44] are well-documented examples of barriers to engagement in HIV and public health, underscoring the need for race-conscious reporting and planned corrective action in EOL research [45].

Within our program at UC San Diego, efforts to advance JEDIA are ongoing. We are pursuing more intentional study design, monitoring, and community-guided refinement. Locally and nationally, PWID and TGD people shoulder disproportionate HIV risk yet rarely appear in HIV cure-related research cohorts; our focus, therefore, is where outreach, trust, support and safety break down for these groups and how to correct those breaks in design and reporting.

#### 2.1.2. Addressing Social Determinants of Health (SDOH) as Research Barriers

Social and structural barriers influence who can participate in research. Factors such as housing instability, transportation challenges, rural distance, and limited language comprehension routinely restrict access to both care and research opportunities [46]. These constraints intensify at the EOL when energy and caregiver capacity are limited [1].

These barriers are not experienced equally across groups. PWID often face stigma, criminalization, and exclusion from clinics and research. TGD individuals may also experience stigma and challenges with health insurance. People without stable housing express severe hardship in handling medical documentation, medication storage, and reliable contact [47,48]. Emerging studies consistently link homelessness and housing instability with poorer engagement and lower odds of viral suppression, highlighting constraints that can also hinder EOL research access [49,50]. From an intersectional lens, concerns about confidentiality and data handling are especially significant for PWID experiencing housing instability, which can ultimately deter participation [51,52]. In the absence of anticipatory design, these structural conditions may reduce opportunities to learn about ongoing studies, limit exposure to recruitment pathways, and make participation unfeasible.

Information flow also determines who learns about studies early enough to consider them. People who are White, English-speaking, and closely connected to clinic networks more often hear about legacy and tissue donation opportunities through clinicians and peers, whereas many Black and Hispanic/Latino participants, women, TGD people, and those with limited English proficiency have fewer trusted points of contact or fewer early conversations about research [53]. Recent qualitative studies with long-term survivors of HIV in the U.S. show that legacy motivations coexist with concerns about burdens and logistics, underscoring the need for designs that proactively address SDOH to enable equitable participation [54,55]. Seemingly neutral eligibility criteria and workflow choices can gatekeep participation when they fail to account for social and structural barriers, effectively favoring individuals with greater resources, even when interest is high [56].

Evidence from HIV care shows that implementing patient-navigation resources improves key engagement outcomes in underserved populations. If we adapt this to research, changes could be reflected in how research teams manage scheduling, transportation, and caregiver support [57]. Specific to PWH, navigation may also include harm-reduction informed engagement, flexible contact methods, and low-threshold scheduling that accounts for shelters, encampments, or inpatient/respite stays.

In the Last Gift program, we are continuing to refine our practices to reduce participation barriers. This has resulted in manuscripts encouraging person-centered approaches and community partnerships to support equitable EOL research participation [4]. Other sites can operationalize the same principle by mapping local recruitment pathways and adding feasible supports such as flexible visit locations, low-threshold scheduling, language access, and navigation through locally trusted clinical and community partners. One effective strategy has been to engage prospective participants during hospital stays, allowing informed consent and follow-up to take place in settings where participants have ready access to the research team. Outside of hospitalizations, more work is needed to address transportation and other SDOH. These findings echo into broader HIV prevention and care research [19,29,42,43,48], underscoring that no study can be truly inclusive without directly addressing SDOH.

### 2.2. Establish Ongoing Consent

#### 2.2.1. Supporting Ethical, Informed Decision-Making

Informed consent functions best when treated as an ongoing process rather than a one-time event [6]. To address potential acquiescence and authority bias, evidence supports the use of extended discussions, teach-back, and multimedia or visual aids for improved understanding, compared to standard written forms [58,59]. These strategies help operationalize equity by ensuring comprehension is less dependent on cognitive or sociocultural background.

At enrollment, participants may designate a legally authorized representative (LAR) (e.g., healthcare proxy, durable power of attorney for health care) to make healthcare-related decisions required for research procedures (e.g., tissue donation) if the participant loses decision-making capacity or if the governing documents permit concurrent decision-making. An assessment of medical decision-making capacity is one strategy to establish ongoing consent at key timepoints and at regular intervals. These can be implemented, for example, before any new research procedure, during routine research encounters, and whenever the participant or proxy requests a capacity assessment.

When capacity is present, confirmation can be obtained directly from the participant; when capacity is lost, decisions are made by the LAR or surrogate. To safeguard participant autonomy, living wills and advanced directives can be used to document preferences regarding autopsy and tissue donation. This helps ensure decisions do not diverge from participant wishes. Each assessment and the source of consent are documented in the research file and, based on institutional policy, in the medical records.

#### 2.2.2. Distinguishing Motivations, Compensation, Reciprocity, and Psychosocial Outcomes

In EOL HIV cure-related research, four related concepts often get blurred: altruistic motivations, compensation, reciprocity, and participant-reported psychosocial outcomes [60]. Altruism frequently motivates participation but is not a study benefit [60]. Compensation (reimbursement or payment) is likewise not a benefit; it offsets time, burden, and expenses and must avoid undue influence [61]. Reciprocity refers to how teams acknowledge contributions (for example, next-of-kin/loved ones-approved memorial options and plain-language updates on how donations informed science) as well as transparent stewardship of data and specimens after death. Psychosocial outcomes such as a sense of dignity, meaning, legacy, or comfort may be experienced by some participants and next-of-kin/loved ones, but they vary by person and must not be promised [62].

In the Last Gift program, we maintain these distinctions, using motivations, compensation, reciprocity, and participant-reported outcomes to guide informed consent language and next-of-kin/loved one’s communications. The goal is clarity and respect: participants’ contributions are honored, burdens are mitigated, and any psychosocial benefits are acknowledged when they arise, without presenting them as guaranteed outcomes of the study. EOL arrangements (for example, cremation) are discussed as personal and logistical choices, or necessities, rather than as study benefits and must never be framed in ways that could pressure enrollment [6]. We recognize that the ability to make EOL arrangements varies across intersecting social and structural factors (for example, housing stability, resources, cultural and religious practices), and study implementation should anticipate and accommodate these differences in planning and communication.

#### 2.2.3. Managing End-of-Life Care Expectations

It is recommended to establish clear role boundaries and communications which separate research from clinical services: clinical teams (primary, hospice, and palliative care) lead symptom control and goal-concordant care while research teams focus on study aims, procedures, and risks. This distinction may be reinforced in scripts and materials, especially at the EOL when research may be conflated with or seen as a pathway to care [6]. Given that research activities are often coordinated with bedside teams, they should never delay analgesia, privacy, or comfort measures; it is recommended to document any care–research tension along with its resolution.

Palliative research ethics, including takeaways from the Statin Study conducted by the Palliative Care Research Group, offer practical safeguards for minimizing burden, protecting privacy, and documenting continuing consent [32]. If not mandated by the presiding Institutional Review Board (IRB), it is recommended that consent language state that medical care is available regardless of enrollment. In practice, we observed reduced therapeutic misconception among participants in Last Gift through clear role boundaries, proxy involvement, repeated consent, and community-guided ethics and lessons learned reports [4].

### 2.3. Center Lived Experiences

#### 2.3.1. Implementing Community-Centered Study Approaches

Centering lived experiences means meaningful engagement through co-design with community advisors, periodic review of accrual and informed consent quality by demographic strata, and documented adjustments when gaps persist, consistent with GPP [39]. This also requires respect for diverse kinship structures—for example, the chosen families of sexual and gender minorities (LGBTQIA+)—in matters of consent, communication, and bedside presence [63]. Engaging participants and proxies as collaborators, and with terminology guided by UNAIDS, enhances accessibility near the EOL by ensuring language concordance, literacy-appropriate materials, and understanding of when home or hospice-based interactions are appropriate [37,38,64,65].

#### 2.3.2. Building Community Relationships

Evidence from HIV community-engaged research shows that partnering with trusted community groups improves access and retention [18,29,30]. These lessons translate to EOL settings when teams co-design approaches with hospice programs, safety-net clinics, and community advisory groups to match local needs [6]. Equally importantly, research teams should close the communication loop by sharing study progress and results back to the communities most affected by HIV, including plain language updates, feedback sessions, and accessible materials that support ongoing dialogue and accountability.

As informed by palliative research ethics, participants can choose where and when procedures occur, who is present, and how updates are shared without compromising scientific quality [35]. Sustained ties with harm-reduction programs, shelter/respite care, soup kitchens, syringe-service programs, and TGD-led community groups improve bidirectional information flow and make customized, low-burden participation possible [20,51,63].

In the Last Gift program, we partner with community and hospice organizations serving underrepresented populations to strengthen information flow and referral pathways, and we strive to maintain contact with families during and after bereavement [4]. In other settings, teams can apply the same approach by establishing locally appropriate partnership agreements, communication norms, and feedback loops with trusted community organizations and care providers. Building and sustaining these relationships demands humility, reciprocity, and transparency. In EOL research, trust is not granted once but renewed through every interaction with participants, their next-of-kin/loved ones, and community partners. Attentive relationship-building transforms research from a transaction into an act of shared stewardship, ensuring that scientific advancement is matched by care and accountability.

#### 2.3.3. Respecting Cultural and Spiritual Considerations

Culture and faith shape how people understand illness, dying, and the donation process [66,67] and can influence their willingness to join EOL research. Evidence linking clinician-supported spiritual care with better quality of life near the EOL suggests that spiritual considerations should be an element of rigorous study design, not a courtesy [34]. Engagement with PWH highlights how each aspect of an individual’s intersectional identity shapes trust and expectations regarding research participation. Respectful practice begins with asking and documenting what matters to each participant [36]. Additionally, historical cases of exploitation, such as the case of Henrietta Lacks, continue to influence how communities perceive research and reinforce the need for transparent stewardship and bidirectional communication [68].

Depending on resources, teams may note and accommodate spiritual and cultural preferences at enrollment. These include offering chaplaincy or culturally concordant support, designating a point person to coordinate with next-of-kin/loved ones, clarifying how privacy and post-death stewardship will be protected, and allowing flexibility in timing, location, and presence to support dignity and ritual needs [64]. When religious or cultural beliefs call for keeping the body intact, participation may still be possible through non-invasive approaches (e.g., ante-mortem surveys or blood draws only) and limited or opt-out post-death procedures [31,69].

Within the Last Gift program, we recognize the room for growth and remain attentive to cultural and spiritual needs. Ultimately, honoring cultural and spiritual dimensions of dying is inseparable from ethical research conduct. Each interaction, whether a conversation about rituals, a moment of silence, or a decision to decline a procedure, reaffirms that participants are research partners, not subjects. Attunement to these values builds the trust that sustains the science and humanity of EOL HIV cure-related research.

Table 1 aligns the three themes of intersectionality-informed EOL HIV cure-related research with actionable proposed steps for implementation. Each practice links ethical intent to measurable behavior, helping research teams monitor inclusion, accessibility, and trustworthiness while protecting participant dignity and advancing scientific rigor. The checklist is designed to be iterative, encouraging phased adoption, reflexivity, and adaptation to local cultural, clinical, and logistical contexts. Study teams can tailor dashboard variables to study aims, local epidemiology, and available resources, define access and corrective action ownership with CAB and ethics input as appropriate, and apply IRB guided safeguards such as aggregating or suppressing small cell sizes to reduce inadvertent identifiability.

#### 2.3.4. Implications Beyond UC San Diego’s Last Gift Program

Phased and reflexive adoption of the proposed steps outlined above can broaden inclusion across intersecting identities, including race, ethnicity, sexual orientation, gender, substance use, and housing status, thereby enhancing external validity and the policy relevance of findings. Embedding intersectionality-informed approaches into routine operations can also improve data completeness (for example fewer missing demographic variables and clearer accrual pathways) and ethical legitimacy (for example clearer role boundaries and ongoing consent), which can strengthen community trust and sustain participation. At the same time, we acknowledge that some recommendations may be resource dependent, including elements that rely on academic medical center infrastructure such as integrated clinical research teams, rapid specimen handling and biorepository capacity, or specialized services, as well as those that require specialized funding for dedicated staff time, navigation and support services, and robust ethics and oversight activities. Programs may also face practical tradeoffs, including balancing staff capacity with multi-session and ongoing consent processes, budget limitations with navigation and support services, and protocol standardization with culturally responsive tailoring. Other components are more transferable across settings, including routine collection and reporting of demographic and social determinants measures, staff training on respectful communication and stigma reduction, standardized consent processes with ongoing check ins, and intentional partnership building with local community organizations. Even modest implementation aligns with emerging expectations from funders and regulators [70] for transparent reporting, enabling programs to scale responsibly while tailoring intensity to local capacity and partnership maturity. A pragmatic sequencing approach is to start with low-resource practices and then add higher-resource components as partnerships, infrastructure, and funding mature. For example, in settings with fewer resources or different legal and service infrastructures, a minimum viable approach may include monitoring enrollment patterns using locally feasible variables, using plain language consent with teach back and documented check ins, and building referral pathways through existing clinics, inpatient services, faith-based groups, or informal community leaders when hospice systems, interpreter services, or formal community organizations are limited. As capacity grows, an enhanced approach may add dedicated navigation, expanded language access support, formal CAB structures, and more intensive bidirectional feedback activities. Sites should align these steps with local legal and ethical requirements, including differences in proxy decision making, tissue donation permissions, and data protection policies.

#### 2.3.5. Limitations and Possible Future Directions

This discussion draws on program experience, community dialogue, and descriptive data from a single academic setting rather than a systematic review or comparative studies, which limits causal inference and generalizability. Selection and reporting biases are possible, and several practices have not yet been evaluated across diverse legal, cultural, and service contexts. Our analysis reflects the experiences of people who enrolled and their next-of-kin/loved ones, and we did not systematically capture the perspectives of people who declined or withdrew. As a result, our understanding of barriers to participation remains incomplete. Future work will implement a low-burden, opt-in approach to invite decliners to share brief reasons for declining and, if they wish, short follow-up reflections.

Future work should prioritize rigorous, multi-site evaluation using shared outcome frameworks, standardized reporting of intersectionality-relevant variables, and inclusion of cost and staffing data to assess feasibility. Beyond research, sustained dialogue is needed, along with integration of intersectionality-informed considerations into requests for applications and grant mechanisms. Continued vigilance, advocacy, and team reflexivity must accompany implementation so that equity aims remain visible, resourced, and accountable over time.

## 3. Conclusions

As one illustrative implementation, the UC San Diego Last Gift program is co-designed with community advisors and includes budgeted access support (e.g., transportation, bedside or home visits, interpreters, flexible scheduling) and wrap-around supports (e.g., navigation, caregiver inclusion, memorial or legacy options, and referrals when resources permit). We conduct periodic equity reviews during participant accrual [5,6,9,10,28]. The Last Gift team holds a weekly case review for each participant to identify needs and adapt procedures (e.g., visit timing and location, language concordance, caregiver involvement, and communication preferences), so participation remains feasible and respectful [4]. Community-engaged research enhances relevance and equity when communities participate as partners throughout the research lifecycle, supported by shared decision-making and institutional backing. Trust develops when researchers balance power, communicate bidirectionally, and maintain transparent stewardship [30,71]. Other programs can operationalize the same domains using different workflows depending on local care settings, infrastructure, and community partnership models.

An intersectionality-informed, participant-centered approach to EOL HIV cure-related research is feasible and essential when implemented gradually with adequate resources. Guided by intersectionality, programs can move from description to action by adopting proportionate and transparent practices, monitoring inclusion through community oversight, and designing workflows that avoid undue burden on research teams. This approach upholds dignity, builds trust, and improves the generalizability and translational value of findings. Embedding intersectionality is not ancillary ethics; it is methodological rigor. It prevents biased science, strengthens accountability, and helps ensure that HIV cure discovery remains worthy of the communities it seeks to serve.

## Figures and Tables

**Table 1:** Preliminary Practice Checklist dashboard for Intersectionality-Informed EOL HIV Cure-Related Research.

Themes	Domains	Aims	Why It Matters	Possible Ways to Implement
1. Facilitate Equitable Access *	Acknowledging equity gaps *	Align participation with who is most affected locally and make course correction accountable	Prevents skewed evidence and focuses action on underrepresented groups with transparency	Match cohort to local HIV epidemiology. Name where inequity shows up. State specific corrective steps and who owns them. Example metric(s): Racial/ethnic enrollment table (tally count), number of wraparound supports provided.
	Addressing social determinants of health as critical research barriers *	Reduce structural barriers so interest becomes feasible participation	Converts willingness into enrollment and retention by addressing transport, language, caregiving, housing, and confidentiality needs	Offer navigation (scheduling/transport/caregiver help). Extend referrals beyond clinic to hospice/safety-net/community groups. Track language needs during outreach. Example metric(s): number of navigation services, survey evaluating access and adequate social support.
2. Establish Ongoing Consent	Supporting ethical, informed decision making	Make comprehension verifiable and ongoing through continuing consent	Lowers therapeutic misconception and protects autonomy while accommodating fluctuating capacity	Implement multi-session consent with teach-back at predefined anchors if feasible. Create role scripts that separate research from clinical care; option to include chosen next-of-kin/loved ones and family. Maintain brief continuing consent log. Implement and monitor ongoing process consent over time. Example metric(s): number of teaching methods used, documentation of proxy understanding (Y/N) and potential novel insights following the application of different teaching methods.
	Distinguishing motivations, compensation, reciprocity, psychosocial outcomes	Maintain ethical clarity while acknowledging contributions	Avoids undue influence and mislabeling of benefits while respecting personhood	Reimburse time/burden (not a benefit). Offer non-material gratitude. Do not promise psychosocial outcomes. Example metric(s): Document participant understanding and motivations for study participation.
	Managing end-of-life care expectations	Keep research and clinical roles distinct while centering comfort and privacy	Protects dignity and prevents role confusion especially near end of life	Use plain language purpose/alternatives; decouple services from enrollment. Coordinate with bedside team so research does not delay symptom relief. Document any care research tension and its resolution and state post-death stewardship. Example metric(s): Document verbal discussion and patient understanding of who to go to for clinical care vs. research, number of clinical care/non-study-related questions directed to study team by mistake
3. Center Lived Experiences *	Implementing community-centered study approaches *	Honor dignity, meaning, and legacy while sharing power in decisions	Centers what matters at the end of life and strengthens trust and procedural fit	Fit procedures to person and next-of-kin/loved one context. Document communication preferences. Offer grief/spiritual supports and reflect legacy goals in memorial options. Example metric(s): Languages/versions of materials aligns with language spoken and culture in the population of PWH.
	Building community relationships *	Build trust before recruitment, throughout participation, and through bereavement	Improves access, retention, and bidirectional accountability	Maintain partnerships with hospice/safety-net/community groups. Agree on communication norms and allow participant-led choices on where/when/who for procedures. Example metric(s): number of ongoing events/initiatives with community groups, survey on satisfaction with study participation (not quality of life or clinical care).
	Respecting Cultural and Spiritual Considerations *	Align participation with cultural and faith needs using a named point person	Reduces distress and supports goal-concordant participation that honors values	Record spiritual/cultural preferences at enrollment. Offer chaplaincy/culturally concordant support with a named point person. Build flexibility into timing/location/presence. Example metric(s): number of cultural and spiritual resources and materials available to participants and proxies.

PWH: People with HIV. * Intersectionality priority: These themes and domains explicitly require structural analysis of interlocking systems and social determinants that shape access, consent, and participant experience.

## Data Availability

Not applicable. No new datasets were generated or analyzed for this commentary.

## Abbreviations

ART	Antiretroviral therapy
PWH	People with HIV
IAS	International AIDS Society
EOL	End of life
HIV	Human Immunodeficiency Virus
UCSD	University of California San Diego
TAG	Treatment Action Group
PWID	People who inject drugs
TGD	Transgender and gender-diverse
JEDIA	Justice, equity, diversity, inclusion, and accessibility
U.S.	United States
CAB	Community Advisory Board
GPP	Good Participatory Practice
UNAIDS	Joint United Nations Programme on HIV/AIDS
SDOH	Social determinants of health
IRB	Institutional Review Board
BIPOC	Black, Indigenous, and other people of color
LGBTQIA+	Lesbian, gay, bisexual, transgender, queer or questioning, intersex, asexual, and related identities
